# Age related endocrine patterns observed in polycystic ovary syndrome patients vs. ovulatory controls: descriptive data from a university based infertility center

**DOI:** 10.1590/2359-3997000000215

**Published:** 2016-09-26

**Authors:** Batool Hossein Rashidi, Mansoureh Gorginzadeh, Soroush Aalipour, Eric Scott Sills

**Affiliations:** 1 Tehran University of Medical Sciences Tehran Iran Vali-e-Asr Reproductive Health Research Center, Tehran University of Medical Sciences, Tehran, Iran; 2 Center for Advanced Genetics Carlsbad California USA Reproductive Research Section, Center for Advanced Genetics, Carlsbad, California, USA; 3 University of Westminster London UK Molecular and Applied Biosciences Department, Faculty of Science & Technology, University of Westminster, London, UK

**Keywords:** Anti-Müllerian hormone, polycystic ovary syndrome, androgens, age

## Abstract

**Objective:**

To compare serum anti-Müllerian hormone (AMH) and other endocrine parameters between patients diagnosed with polycystic ovary syndrome (PCOS) and age-matched ovulatory women.

**Materials and methods:**

AMH, DHEAS, FSH, LH, PRL, TSH and total testosterone (TT) were prospectively measured in oligo-ovulatory PCOS patients (*n* = 595) and in ovulatory non-PCOS women (*n* = 157) referred to a tertiary infertility center. Mean BMI was similar across the two study populations and there were no smokers in the sample. Patients in both groups were further classified into three categories by age: < 25 yrs, 25-34 yrs, and ≥ 35 yrs. Selected clinical and demographic characteristics were tabulated for each group.

**Results:**

Serum AMH was significantly higher among PCOS patients compared to non-PCOS controls in the non-stratified sample (7.54 ± 5.8 vs. 2.49 ± 2.0 ng/mL, respectively; p < 0.0001), while serum FSH, DHEAS, TSH and prolactin were similar for both groups (*p* > 0.05). As expected, mean (total) testosterone levels were notably different between PCOS vs. non-PCOS controls (0.84 ± 0.76 vs. 0.43 ± 0.38 ng/mL, respectively; *p* < 0.001), and mean AMH level was significantly lower in the oldest age category (> 35 yrs) compared to both younger control groups (*p* < 0.0001). Both DHEAS and total testosterone decreased with age among PCOS patients, although mean serum DHEAS for women age > 35 yrs was significantly lower than DHEAS measured in younger women with PCOS (*p* < 0.02). For PCOS patients, AMH remained relatively stable irrespective of age.

**Conclusion:**

Although AMH can serve as a satisfactory marker of ovarian reserve, for PCOS patients the expected decline in AMH associated with reproductive aging appears attenuated despite ovarian senescence. In contrast, mean DHEAS levels were markedly lower among older PCOS women (> 35 yrs) compared to younger PCOS patients.

## INTRODUCTION

Since the introduction of anti-Müllerian hormone (AMH) as a method of estimating ovarian reserve, its measurement in the setting of the advanced reproductive technologies has received considerable attention. As a proxy marker for ovarian granulosa cell activity (or antral follicle count), serum AMH has also proven useful in prediction of poor responders and ovarian hyperstimulation syndrome (OHSS) in IVF cycles (1,2). The association between AMH and the pathogenesis of polycystic ovary syndrome (PCOS) dates from 1997, when AMH was first measured in serum and follicular fluid of women with PCOS and tubal factor infertility undergoing IVF (3). Given the multicystic ovarian stroma commonly present in PCOS, it is not surprising that relatively higher serum AMH levels in PCOS patients compared to age- and weight-matched ovulatory (*i.e*., non-PCOS) controls have been observed, suggesting its potential use as a diagnostic tool for PCOS. Accordingly, several threshold AMH values for PCOS (based on measurements among varied age and ethnic groups) have been proposed. Of note, serum AMH has been considered as a substitute for antral follicle count as articulated by the Rotterdam diagnostic criteria for PCOS (4-12) although this has not been widely used perhaps due to the lack of a universally standardized AMH serum assay (13).

Serum AMH measurements may help differentiate between polycystic ovary morphology (PCOM) and PCOS; it has been suggested that PCOM (once regarded as a normal variant of ovarian morphology) actually represents a precursor to the full manifestation of PCOS (14). As phenotypic features and endocrine profiles of PCOS patients can change over time (15), the classic pattern of hyperandrogenism and oligo-ovulation may not necessarily be present in older patients. Accordingly, the Rotterdam criteria may not be suited for all women with PCOS and the application of an age adjusted criteria would be useful (16). The current investigation reports on serum AMH levels and other endocrine parameters observed among PCOS women referred for reproductive endocrinology evaluation at our center, and contrasts these findings with normal ovulatory (non-PCOS) patients evaluated at the same institution.

## MATERIALS AND METHODS

This prospective, observational matched-cohort study assessed all referrals to the reproductive endocrinology clinic of the Vali-e-Asr Research Center in Tehran, Iran between March 2014 and April 2015. Approval from the Tehran University of Medical Sciences human subjects research panel was obtained before study launch, and all participants provided written informed consent at entry. To enroll patients in the PCOS group, the Rotterdam criteria (16) were used to screen for affected women. Patients with a diagnosis of cancer (gynecologic or non-gynecologic), psychiatric illness, neurologic disease, diabetes, chronic pelvic pain, endometriosis or a debilitating medical condition were excluded. Women greater than age fifty were also not included in this analysis. Basic demographic characteristics were retrieved from our medical records database and tabulated with each patient’s test results for serum FSH, LH, PRL, AMH, DHEAS, TSH, testosterone, as well as hemoglobin and hematocrit. Hormone and peptide levels were determined by standard radioimmunoassay in accordance with manufacturer guidelines (Abbott Laboratories S.A.; Dubai, UAE). For this investigation, a total of 752 subjects were evaluated from a single institution during the 14 month study interval and were further classified into three categories by age: < 25 yrs, 25-34 yrs, and ≥ 35 yrs.

### Statistical analysis

Data were analyzed using student’s *t*-test, Chi-square, or one-way ANOVA as appropriate. Threshold values for AMH and LH as predictors of PCOS were determined from a receiver operating characteristic (ROC) curve. Correlation and logistic regression analyses were performed to assess associations between clinical and demographic parameters.

## RESULTS

**Figure 1:**
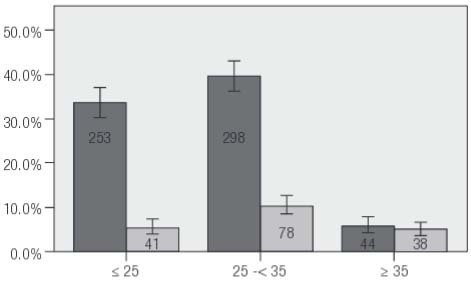
Number and percent distribution of study patients (PCOS, *dark grey*; non-PCOS, *light grey*) by age classification (CI 95%) encountered during the 14 month research interval.

During the 14 month study interval, a total of 3,512 unique patient encounters were registered at our institution; patients meeting standard PCOS criteria accounted for 595 of these visits (16.9%) and all volunteered for study enrollment. Clinical and demographic data from these individuals were compared with findings from healthy, ovulatory (non-PCOS) controls evaluated during the same interval (*n *= 157). Patient age distribution for those who enrolled in this study is presented in Figure 1. Mean ± SD age for PCOS patients was significantly lower than for control patients during the study period (26.9 ± 4.6 vs. 29.9 ± 5.9 yrs; *p *= 0.12), although mean BMI was similar in the two groups (*p *> 0.05). Perhaps not surprisingly given this age difference, mean ± SD AMH level was 7.54 ± 5.78 ng/mL for women with PCOS and was 2.49 ± 4.45 ng/mL among non-PCOS controls (*p *< 0.001). A summary of values measured for all study parameters for patients in both groups is given in Table 1.

**Table 1:** Summary of clinical features and laboratory data for 752 women enrolled during the 14-month study interval

	PCOS	non-PCOS	***P^1^***
*n*	595	157	
Age (yrs)	26.94 ± 4.57	29.96 ± 5.91	< 0.001^b^
BMI (kg/m^2^)	22.27 ± 4.91	25.83 ± 4.45	0.118
Infertility duration (yrs)	4.62 ± 3.58	5.92 ± 4.86	0.002
Cycle duration (d)	45.74 ± 22.55	31.55 ± 7.61	< 0.001
AMH (ng/mL)	7.54 ± 5.78	2.49 ± 1.96	< 0.001
FSH (IU/L)	6.00 ± 3.14	6.29 ± 3.28	0.236
LH (IU/L)	9.03 ± 6.91	4.41 ± 2.67	< 0.001
DHEAS (μg/dL)	139.69 ± 87.8	117.56 ± 82.9	0.581
Testosterone – total (ng/mL)	0.84 ± 0.76	0.43 ± 0.37	< 0.001
Prolactin (ng/mL)	14.75 ± 11.79	15.25 ± 11.4	0.803
TSH (IU/L)	2.48 ± 1.72	2.16 ± 1.54	0.082
Hemoglobin (g/dL)	13.13 ± 1.21	12.92 ± 1.0	0.053
Hematocrit (%)	39.71 ± 3.16	39.39 ± 2.75	0.310

All data reported as mean ± SD. ^1 ^by Student’s *t-*test.

**Figure 2:**
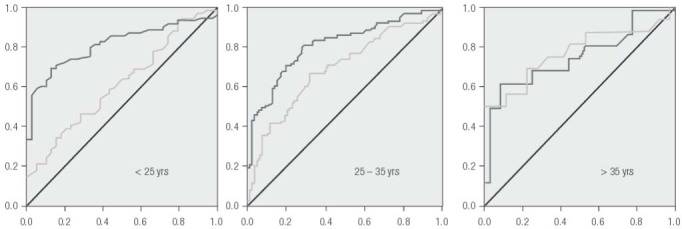
Receiver operating characteristic (ROC) curves for serum AMH (*dark grey*) and LH (*light grey*) calculated from levels measured in a referred population of infertility patients stratified by age, where Y axis = sensitivity, X axis = specificity.

**Figure 3:**
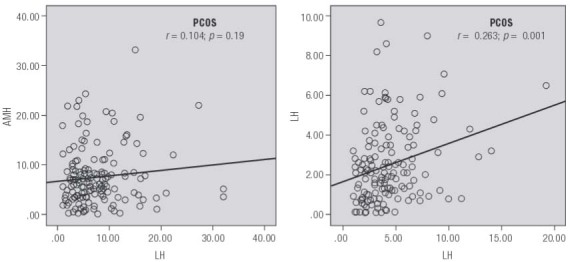
Correlation analysis for serum AMH and LH levels measured during a 14-month study period among study patients (*n* = 752). For PCOS and control patients, R2 linear = 0.011 and 0.069, respectively (by Pearson’s *r* ).

Using these data, ROC curves were developed to evaluate the diagnostic capacity of AMH for PCOS as a function of age (Figure 2). In our population the AUC of AMH levels was 0.826 (95% CI = 0.780-0.872) and the optimal AMH threshold (diagnostic cut-off) level was determined to be 3.77 ng/mL, yielding 72.8% sensitivity and 78.4% specificity. Indeed, serum AMH had higher sensitivity and specificity for the diagnosis of PCOS compared to serum LH. Of note, we observed a significant correlation between serum AMH and LH measurements in the non-PCOS group (Figure 3).

**Figure 4:**
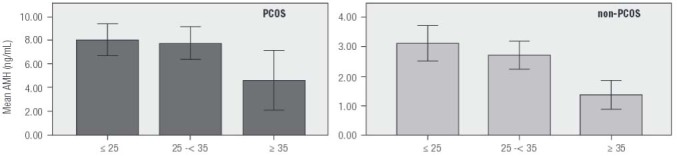
Serum AMH levels prospectively measured in patients with PCOS (*n* = 595) and non-PCOS controls (*n* = 157) stratified by age during a 14 month clinical study period (CI 95%).

Serum AMH levels for both patient groups were next stratified into three levels, < 2 ng/mL, between 2-5 ng/mL, and > 5 ng/mL, and evaluated. This classification revealed that most patients in the PCOS group had the highest serum AMH measurements, and patients in the control group (non-PCOS) had the lowest. Patients were further partitioned into three age categories (< 25, 25-34, ≥ 35 yrs) and Figure 4 summarizes this distribution.

**Figure 5:**
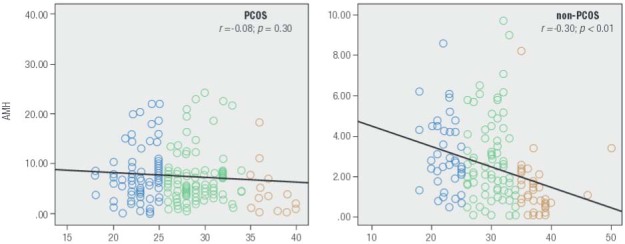
Age-related changes in serum AMH (ng/mL) as measured prospectively in infertility patients (*n* = 752) attending for assessment during a 14 month study period. While longitudinal declines in AMH were measured in both populations, this change was significant only in the non-PCOS control group.

While serum AMH levels varied inversely with patient age, this downward trend was significant only among non-PCOS patients in our sample (Figure 5). Mean serum AMH level for patients age ≥ 35 yrs in the non-PCOS group was significantly lower than for those younger than age 25 yrs (*p *< 0.001) and those between age 25-35 yrs (*p *= 0.002). Moreover, a significant linear correlation was observed between serum AMH and age among non-PCOS control patients (*r* = -0.303; *p *< 0.001). There was also a significant difference between mean serum total testosterone levels for women in the PCOS group and the non-PCOS group (0.84 ± 0.76 vs. 0.43 ± 0.38 ng/mL, respectively; *p *< 0.001). The difference was not significant for serum DHEAS, however (*p *= 0.581). For PCOS women, mean serum levels for both DHEAS and total testosterone declined with age, although the difference between age groups was not significant for total testosterone (*p *= 0.316). Mean serum DHEAS level for women age > 35 was significantly lower than for among those < 25 yrs and those between age 25-35 yrs (*p *= 0.003 and 0.02, respectively). For non-PCOS (control) patients, mean total testosterone tended to decrease with age, although this reduction was not significant between any two age categories (*p *= 0.155). There were also no important differences in DHEAS levels when any two age groups were compared (*p *= 0.322). Thus despite the apparent downward trends, the observed values for these two androgens did not change significantly with aging. While we noted a significant correlation between serum testosterone level and hemoglobin concentrations in the non-PCOS group, this correlation was not significant for the PCOS group.

To evaluate the relation between BMI and AMH, we categorized study subjects into two groups: those with BMI < 25 kg/m^2^ and those with BMI ≥ 25 kg/m^2^. Mean serum concentrations of serum AMH along with total testosterone and DHEAS were not significantly different for these two BMI classes in the PCOS group (*p *> 0.70). Likewise, there was no significant correlation between serum AMH and BMI in this group (r = -0.125; *p *= 0.123). However, for study subjects in the non-PCOS group, BMI > 25 was associated with significantly higher serum total testosterone (0.49 ± 0.46 vs. 0.34 ± 0.22 ng/mL; *p *= 0.039). This association was not observed for serum DHEAS or AMH in our study population.

## DISCUSSION

This investigation aimed to provide additional data on PCOS and contribute an improved understanding to the process of how serum AMH changes with increasing age in PCOS. Such an effort would seem relevant as AMH has been suggested as a marker useful in the diagnosis of PCOS (14). Although PCOS is the most common hormonal disorder among reproductive age women (17), is the leading cause for anovulatory/oligoovulatory infertility (18), and has the potential for serious long-term health effects (19,20), there remains no universal agreement on how the condition is diagnosed (21). Thus, clarification of serum AMH and its longitudinal patterns could assist clinicians in the evaluation of patients suspected of having PCOS. Indeed, thus far there has been no place for the possible impact of age in the current diagnostic criteria for PCOS. This circumstance prevails even though the clinical and biochemical presentations of PCOS are known to change over time, as patient age increases (15).

Considering the relatively high prevalence of PCOS in south-East Asian populations (22) coupled with the increased incidence of infertility patients of advanced maternal age (15), the central objective of this research was to report on observed variations in AMH levels as a function of aging, both in PCOS and non-PCOS patients. Our findings offer additional support for a significant inverse correlation between AMH and age in healthy, ovulatory women, expected as ovarian reserve diminishes with the process of normal reproductive aging (1,2). However, the fact that this decrease was not observed among women with PCOS represents a novel finding. In other words, mean levels of serum AMH did not significantly diminish even as reproductive aging progressed. This observation was in contrast to DHEAS levels, which did significantly decrease in older patients. Consequently, as proposed previously (15) serum AMH might provide improved diagnostic accuracy compared to traditional Rotterdam criteria. Our data suggests this may be particularly useful for older (*i.e.,* > 35 yrs) PCOS women, as this approach could offer superior capacity compared to standard serum total testosterone and DHEAS measurements (15,16).

One of the challenges of prospective clinical research investigation is the inability to control for specific demographic and clinical features of the patients who present during the study interval. At our facility, the mean age of the PCOS patients was significantly different than the age of the control (non-PCOS) group, which represents a limitation of our study. However, this issue was addressed by the partitioning of these two patient groups into three standard age classes, and permitted the development of a serum AMH threshold level useful in assessing PCOS in this referral population in Iran. This study could have been strengthened by a more detailed tabulation of each PCOS patient’s clinical presentation with respect to the Rotterdam criteria, although this was beyond the scope of our investigation.

Our results, based on observations from > 750 infertile women referred to a tertiary infertility unit here, show how serum AMH could be a useful parameter in the diagnosis of PCOS. Of note, the proposed diagnostic “breakpoint” value for AMH at 3.77 ng/mL calculated from our clinical population is intermediate to those reported earlier by Sahmay and cols. (3.8 ng/mL) and Wiweko and cols. (4.45 ng/mL) (4,5). In contrast with results published by Kriseman and cols. (23), BMI was not significantly associated with serum AMH levels in our PCOS population. One possible explanation for this finding is that our PCOS patients were younger and leaner compared to other PCOS populations, or perhaps the impact of age exerts a greater effect than does BMI on AMH. While this evaluation explores some of the constellation of possible ramifying factors which influence serum AMH, more studies on large and diverse populations will be needed.

## References

[1] .Seifer DB, MacLaughlin DT. Mullerian inhibiting substance is an ovarian growth factor of emerging clinical significance. Fertil Steril. 2007;88(3):539-46. 10.1016/j.fertnstert.2007.02.014 17559842

[2] .De vet A, Laven JS, De Jong FH, Themmen AP, Fauser BC. Antimullerian hormone serum levels: a putative marker for ovarian ageing. Fertil Steril. 2002;77:357-62. 10.1016/s0015-0282(01)02993-4 11821097

[3] .Fallat ME, Siow Y, Marra M, Cook C, Carillo A. Mullerian inhibiting substance in follicular fluid and serum: a comparison of patients with tubal factor infertility, polycystic ovary syndrome, and endometriosis. Fertil Steril. 1997;67:962-5. 10.1016/s0015-0282(97)81417-3 9130910

[4] .Sahmay S, Aydin Y, Oncul M, Sentark LM. Diagnosis of polycystic ovary syndrome: AMH in combination with clinical symptoms. J Assist Reprod Genet. 2014;31:213-220. 10.1007/s10815-013-0149-0 PMC3933601 24343036

[5] .Wiweko B, Maidarti M, Priangga MD, Shafira N, Fernando D, Sumapraja K, et al. Anti-mullerian hormone as a diagnostic and prognostic tool for PCOS patients. J Assist Reprod Genet. 2014;31(10):1311-6. 10.1007/s10815-014-0300-6 PMC4171421 25119192

[6] .Hwang YI, Sung NY, Koo HS, Cha SH, Park CW, Kim JY, et al. Can high serum anti-Müllerian hormone levels predict the phenotypes of polycystic ovary syndrome (PCOS) and metabolic disturbances in PCOS patients? Clin Exp Reprod Med. 2013;40(3):135-40. 10.5653/cerm.2013.40.3.135 PMC3811721 24179872

[7] .Chun S. Serum luteinizing hormone level and luteinizing hormone/follicle stimulating hormone ratio but not serum anti-Müllerian hormone level is related to ovarian volume in Korean women with polycystic ovary syndrome. Clin Exp Reprod Med. 2014;41(2):86-91. 10.5653/cerm.2014.41.2.86 PMC4102695 25045633

[8] .Bhide P, Dilgil M, Gudi A, Shah A, Akwaa C, Homburg R. Each small antral follicle in ovaries of women with polycystic ovary syndrome produces more anti-müllerian hormone than its counterpart in a normal ovary: an observational cross-sectional study. Fertil Steril. 2015;103(2):537-41. 10.1016/j.fertnstert.2014.10.033 25467043

[9] .Leonhardt H, Hellström M, Gull B, Lind AK, Nilsson L, Janson PO, et al. Ovarian morphology assessed by magnetic resonance imaging in women with and without polycystic ovary syndrome and associations with anti-müllerian hormone, free testosterone, and glucose disposal rate. Fertil Steril. 2014;101(6):1747-56. 10.1016/j.fertnstert.2014.02.022 24661732

[10] .Alebis MŠ, Stojanovic% N, Duhamel A, Dewailly D. The phenotypic diversity in per-follicle anti-Müllerian hormone production in polycystic ovary syndrome. Hum Reprod. 2015;30(8):1927-33. 10.1093/humrep/dev131 26048913

[11] .Casadei L, Madrigale A, Puca F, Manicuti C, Emidi E, Piccione E, et al. The role of serum anti-Müllerian hormone (AMH) in the hormonal diagnosis of polycystic ovary syndrome. Gynecol Endocrinol. 2013;29(6):545-50. 10.3109/09513590.2013.777415 23506275

[12] .Dewailly D, Alebis MŠ, Duhamel A, Stojanović N. Using cluster analysis to identify a homogeneous subpopulation of women with polycystic ovarian morphology in a population of non-hyperandrogenic women with regular menstrual cycles. Hum Reprod. 2014;29(11):2536-43. 10.1093/humrep/deu242 25267785

[13] .Marron KD, Sills ES, Cummins PL, Harrity C, Walsh DJ, Walsh APH. Impact of pre-mixing AMH serum samples with standard assay buffer: ovarian reserve estimations and implications for clinical IVF providers. J Reprod Endocrinol Infertil. 2016;2:10.

[14] .Homburg R, Ray A, Bhide P, Gudi A, Shah A, Timms P, et al. The relationship of serum anti-Mullerian hormone with polycystic ovarian morphology and polycystic ovary syndrome: a prospective cohort study. Hum Reprod. 2013;28(4):1077-83. 10.1093/humrep/det015 23377771

[15] .Kushnir VA, Halevy N, Barad DH, Albertini DF, Gleicher N. Relative importance of AMH and androgen changes with aging among non-obese women with polycystic ovary syndrome. J Ovarian Res. 2015;8(1):45. 10.1186/s13048-015-0175-x PMC4496928 26156856

[16] .Lauritsen MP, Bentzen JG, Pinborg A, Loft A, Forman JL, Thuesen LL, et al. The prevalence of polycystic ovary syndrome in a normal population according to the Rotterdam criteria versus revised criteria including anti-Mullerian hormone. Hum Reprod. 2014;29(4):791-801. 10.1093/humrep/det469 24435776

[17] .Cronin L, Guyatt G, Griffith L, Wong E, Azziz R, Futterweit W, et al. Development of a health related quality of life questionnaire for women with polycystic ovary syndrome (PCOS). J Clin Endocrinol Metab. 1998;83(6):1976-87. 10.1210/jcem.83.6.4990 9626128

[18] .Hull MGR. Epidemiology of infertility and polycystic ovarian disease: endocrinological and demographic studies. Gynecol Endocrinol. 1987;1(3):235-45. 10.3109/09513598709023610 3140583

[19] .Solomon CG, Hu FB, Willett WC, et al. History of irregular menstrual cycles and risk for coronary heart disease [abstract #107920]. 73rd Annual Scientific Session, American Heart Association, 1999.

[20] .Kelly CJ, Connell JM, Cameron IT, Gould GW, Lyell H. The long term health consequences of polycystic ovary syndrome. BJOG. 2000;107:1327-38. 10.1111/j.1471-0528.2000.tb11644.x 11117758

[21] .Sills ES, Perloe M, Tucker MJ, Kaplan CR, Genton MG, Schattman GL. Diagnostic and treatment characteristics of polycystic ovary syndrome: descriptive measurements of patient perception and awareness from 657 confidential self-reports. BMC Womens Health. 2001;1(1):3. 10.1186/1472-6874-1-3 PMC55341 11545683

[22] .Bhide P, Gudi A, Shah A, Homburg R. Serum anti-Mullerian hormone levels across different ethnic groups: a cross-sectional study. BJOG. 2015;122(12):1625-9. 10.1111/1471-0528.13103 25286823

[23] .Kriseman M, Mills C, Kovanci E, Sangi-Haghpeykar H, Gibbons W. Anti-mullerian hormone levels are inversely associated with body mass index (BMI) in women with polycystic ovary syndrome. J Assist Reprod Genet. 2015;32(9):1313-6. 10.1007/s10815-015-0540-0 PMC4595400 26238387

